# Unique among high passes: Phylogenetic inferences from DNA barcoding of the butter**fl**y fauna of Ladakh Trans-Himalaya, India

**DOI:** 10.21203/rs.3.rs-4392854/v1

**Published:** 2024-05-20

**Authors:** Mohd Ali, Rushati Dey, Moumita Das, Vikas Kumar, Kailash Chandra, Virendra Prasad Uniyal, Sandeep Kumar Gupta

**Affiliations:** Zoological Survey of India; Zoological Survey of India; Zoological Survey of India; Zoological Survey of India; Zoological Survey of India; Wildlife Institute of India Chandrabani; Wildlife Institute of India Chandrabani

**Keywords:** Cytochrome C Oxidase I, Bayesian analyses, phylogenetic clades, nested lineage, intersubspecific distances, Karanasa

## Abstract

The butterfly assemblage of Ladakh Trans-Himalaya demands a thorough analysis of their population genetic structure owing to their typical biogeographic affinity and their adaptability to extreme cold-desert climates. No such effort has been taken till date, and in this backdrop, we created a barcode reference library of 60 specimens representing 23 species. Barcodes were generated from freshly collected leg samples using the Sanger sequencing method, followed by phylogenetic clade analyses and divergence calculation. Our data represents 22% of Ladakh’s Rhopaloceran fauna with the novel barcode submission for six species, including one Schedule II species, *Paralasa mani*. Contrary to the 3% threshold rule, the interspecific divergence between two species pairs of typical mountain genus Hyponephele and Karanasa was found to be 2.3% and 2.2%, respectively. The addition of conspecific global barcodes revealed that most species showed little increase in divergence value, while a two-fold increase was noted in a few species. Bayesian clade clustering outcomes largely aligned with current morphological classifications, forming monophyletic clades of conspecific barcodes, with only minor exceptions observed for the taxonomically complicated genus *Polyommatus* and misidentified records of *Aulocera* in the database. We also observed variations within the same phylogenetic clades forming nested lineages, which may be attributed to the taxonomic intricacies present at the subspecies level globally, mostly among Eurasian species. Overall, our effort not only substantiated the effectiveness of DNA Barcoding for the identification and conservation of this climatically vulnerable assemblage but also highlighted the significance of deciphering the unique genetic composition among this geographically isolated population of Ladakh butterflies.

## Introduction

Because of their diversity and functional importance in the ecosystem, butterflies serve as valuable indicators for monitoring biodiversity changes [1]. From being pollinators and playing major roles in the complex food web to adding aesthetic values, butterfly surveys have become a potential focal system for ecological monitoring. However, in the era of massive economic over-exploitation and intergovernmental inaction, where global ecological loss has become the norm, the butterfly population continues to decline rapidly, much of which remains unaccounted. In this situation, it is essential to document and quantify the global butterfly fauna before they vanish or shift their range drastically. Due to their complex life cycle, multiple morphs, and regional morphological variations, butterfly identification is a challenge. Moreover, due to the lack of taxonomic expertise, traditional morpho-taxonomy approaches for species identification cannot keep up with the current pace of biodiversity loss. However, over the last two decades, DNA barcoding techniques have been able to reasonably speed up the process of species documentation and biodiversity characterization across different taxa and countries [2–3].

Using the 648 bp Cytochrome C Oxidase *I (COI)* gene as the marker, barcoding has been successful in discriminating species across the animal kingdom [4], including the highly diverse and cryptic insect orders [5–6]. Butterflies being model organisms, many studies have taken advantage of this technique to understand the region-specific butterfly biodiversity [7–12]. Barcodes, coupled with a few other genes, were not only able to successfully resolve the taxonomic quest and crypticism in butterfly systematics [13–14], but also provided valuable insights regarding butterfly phylogeography and ecology [15–16]. Nevertheless, a comprehensive understanding of large-scale patterns necessitates the analysis of numerous specimens [17–18], and the effectiveness of DNA barcoding has hence encouraged the construction of barcode reference libraries for various groups, including butterflies [19–20]. These libraries are crucial for the documentation of biodiversity and also for studying large-scale phylogeographic patterns. Presently, BOLD hosts a database of 163K barcodes for 10,553 named butterfly species, worldwide. However, most of these barcodes are contributions from European, American and African countries, with very few studies from South-East Asia that include barcodes for a significant fraction of the butterfly fauna of Central Asia [7], Pakistan [10], and Malaysia [21]. Coming to the Indian scenario, the integration of molecular tools and traditional morphology for butterfly taxonomy has seen little investigation, with only 601 available barcodes in BOLD representing 166 species. Significant contributions have been made by Gaikwad et al [22] and Singh et al. [23] towards a curated reference library for the butterfly fauna of Western Ghats and Western Himalaya respectively.

India is a megadiverse country, and the Himalayas being one of the world’s biodiversity hotspots provide an array of habitat types, that is home to a cryptic and endemic butterfly fauna. Ladakh, meaning “Land of High Passes”, situated at the confluence of Palearctic and Oriental biogeographic zones, hosts a unique butterfly fauna having affinities both towards Eurasia and Tibetan Himalaya. The peculiar geographical, topographical and climatic conditions of Ladakh have restricted these species assemblages to isolated populations, the high passes acting as barriers between them and shaping their unique genetic composition. This fragile Trans-Himalayan ecosystem is home to 101 species of butterfly [24], including numerous high-altitude, range-restricted and globally threatened butterfly species. For typical mountainous species, genetic diversity often correlates with population connectivity through gene flow [25] and adaptations to local habitat conditions [26]. Some Palaearctic butterfly species, previously subjected to phylogeographic studies, have shown to exhibit distinct genetic characteristics between Europe and Asia [27–28] and despite having continuous species range across Eurasia, certain butterfly species have also revealed distinct European genetic lineages [29]. Therefore, a thorough investigation of the population genetic structures of butterflies in Ladakh is essential to comprehend how historical and ongoing demographic processes influence species distribution patterns in a fragile landscape like Ladakh. However, our knowledge of butterfly habitat preferences in the trans-Himalayan region of Ladakh remains limited, and the natural history of many butterfly groups remains elusive. Even though it’s the need of the hour, no such barcoding efforts have been carried out in this landscape, except for the very few sequences available only for 4 species from Ladakh [30–31]. This knowledge gap not only hampers conservation efforts in this climatically vulnerable landscape but also fails to understand the genetic lineage of the unique Ladakh fauna. Addressing these issues, the current study was designed with the primary goal of building a DNA barcode reference library for the butterfly species of Ladakh and testing the effectiveness of barcode databases in their identification. Moreover, we also compared the generated barcodes in a worldwide scale for a better understanding of the extent of variation present within and between species and subspecies.

## Materials and methods

### Collection and preservation

**Figure 1:**
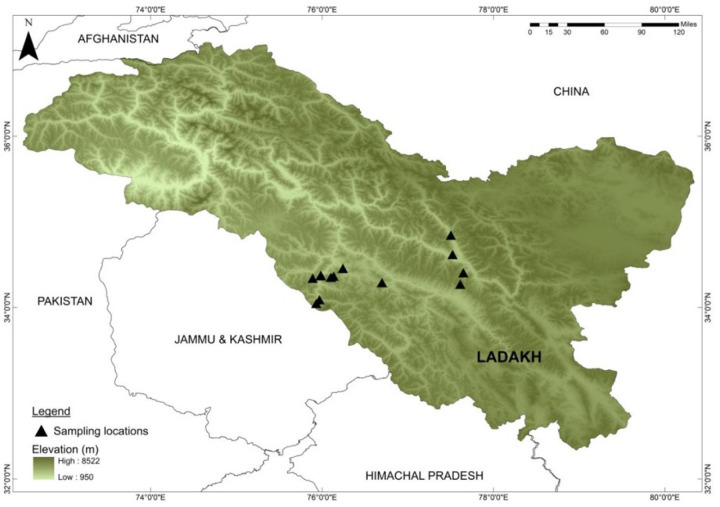
Butterfly sampling locations across the altitudinal gradient of Ladakh Trans-Himalaya, sampled during July-August 2019

This study was conducted in the Trans - Himalayan regions of Ladakh Union Territory of India. Total of 60 samples were collected from different locations of Ladakh (Fig. 1; Table 1) and deposited in the Lepidoptera collections of Zoological Survey of India. Specimens were stretched, pinned, labeled, dried, and preserved in a dry cabinet. Species were identified by observing the wing shape, wing spots, and color patterns described in available keys and identification guidebooks [24, 32].

### DNA extraction, amplification and sequencing

Two or three leg samples were taken out from the morphologically identified specimens with sanitized forceps and stored in molecular grade 70% ethanol at 4 °C. Genomic DNA was extracted from the leg samples following the standard protocol of Phenol Chloroform-Isoamyl alcohol [33]. The primer pair, LepF1: 5’-ATTCAACCAATCATAAAGATATTGG-3’ & LepR1: 5’- TAAACTTCTGGATGTCCAAAAAATCA-3’ was used to amplify the 648 bp barcode region COI (Cytochrome C Oxidase subunit I) of the mitochondrial DNA. PCR was done using 20 μL of Q2 Green PCR Master Mix (Promega, Madison, WI, USA) in a Veriti VR Thermal Cycler (Applied Biosystems, Foster City, CA) with the following thermal cycling profile: first cycle of 5 min at 94 °C, followed by 5 cycles of 1 min at 94 °C, 1 min 30 sec at 45 °C, 1 min 30 sec at 72 °C; followed by 30 cycles of 1 min at 94 °C, 1 min 30 sec at 51 °C, 1 min 30 sec at 72 °C, and final extension for 5 min at 72 °C. After the purification of PCR products using the QIAquick Gel Extraction Kit (Qiagen Inc., Germantown, MD), cycle sequencing was performed with BigDye Terminator ver. 3.1 Cycle Sequencing Kit (Applied Biosystems Inc., California, USA) and finally sequenced using 48 capillary ABI 3730 Genetic analyzer in Zoological Survey of India, Kolkata.

### Sequence quality control measure and phylogenetic analysis

The generated forward and reverse sequences of COI fragments were analyzed in SeqScape software version 2.7 (Applied Biosystems Inc.) and consensus sequences were acquired after checking deletion, insertion, and stop codons. The 60 assembled sequences were aligned using the Multiple sequence alignments performed using the ClustalW multiple alignments function in BioEdit version 7.0 [34]. All the sequences were validated using the BLAST tool in the NCBI (blast.ncbi.nlm.nih.gov/Blast.cgi). It was followed by manual screening and trimming to have a uniform dataset of 610 bp for further analysis. Additionally, 92 barcodes from GenBank were incorporated in the tree to conform to the species identifications (Table S1). Nucleotide compositions and pairwise evolutionary genetic divergences were estimated using the Kimura 2 Parameter (K2P) model with the MEGA11 program [35]. For calculating the divergence between region-specific local populations for a few selected species, their COI sequences were downloaded from BOLD (Barcode of Life Database), sorted and grouped according to collection locality, and aligned using MEGA11, followed by distance calculation using the K2P model. Bayesian phylogenetic inference analysis was run in MrBayes 3.2 [36] using the model generated in jModelTest. The analysis comprised two runs of Markov chain Monte Carlo simulations (MCMC), with flat priors, dataset partitioned by two million generations, sampling every 100 generations with 10% of samples discarded as burn-in. Tree-Annotator v1.8.1 was used to select the maximum clade credibility (MCC) tree [37], which was visualized in FigTree.v1.4.4 [38]. *Apis florea* (Order: Hymenoptera) (GenBank Accession No. MH378769.1) was chosen as an outgroup.

## Results

We successfully generated 612–680 bp DNA sequences from 60 butterfly specimens. These specimens were morphologically classified into 17 genera, spanning 10 subfamilies across five butterfly families. Of these, 57 were identified up to the species level and classified into 22 distinct morpho-species. The remaining three specimens were first assigned to their respective genera (marked as + in Table 1), and was later resolved to species level using molecular taxonomy, thus leading up to barcodes for 23 species. Most species were represented by two or more barcodes, except 8 species by a single barcode. On conducting a similarity search of the generated sequences, using BLASTn in GenBank and Barcode of Life Database (BOLD) identity search, barcodes for 15 morphologically identified species exhibited a robust match with the database sequences and showed high similarity, ranging from 97 – 100%; while barcodes for the remaining 8 species were correctly matched only up to the genus level (Table S2). All the sequences generated in this study, which also includes the novel submissions (marked as * in Table 1) for six species, can be accessed in GenBank under the accession numbers OR600796 - OR600855 (Table 1).

Our sequence analysis revealed 244 variable characters, comprising 245 parsimony informative sites and 381 conserved sites. Nucleotide frequencies were distributed as follows: 30.3% (A), 39.9% (T), 14.4% (G), and 15.4% (C). The base composition exhibited a bias towards Adenine and Thymine, constituting a combined total of 70.2% (Table 2), the composition aligning with typical characteristics of other invertebrate genes. The mean A+T content was found to be 59.5%, 58.4%, and 92.1% in the first, second, and third codon positions of the COI fragment, respectively. In our generated sequences, we observed notable haplotype gene diversity (Hd) of 0.989, nucleotide diversity per site (Pi) of 0.12064, and Tajima’s D statistic of 0.35174.

**Figure 2:**
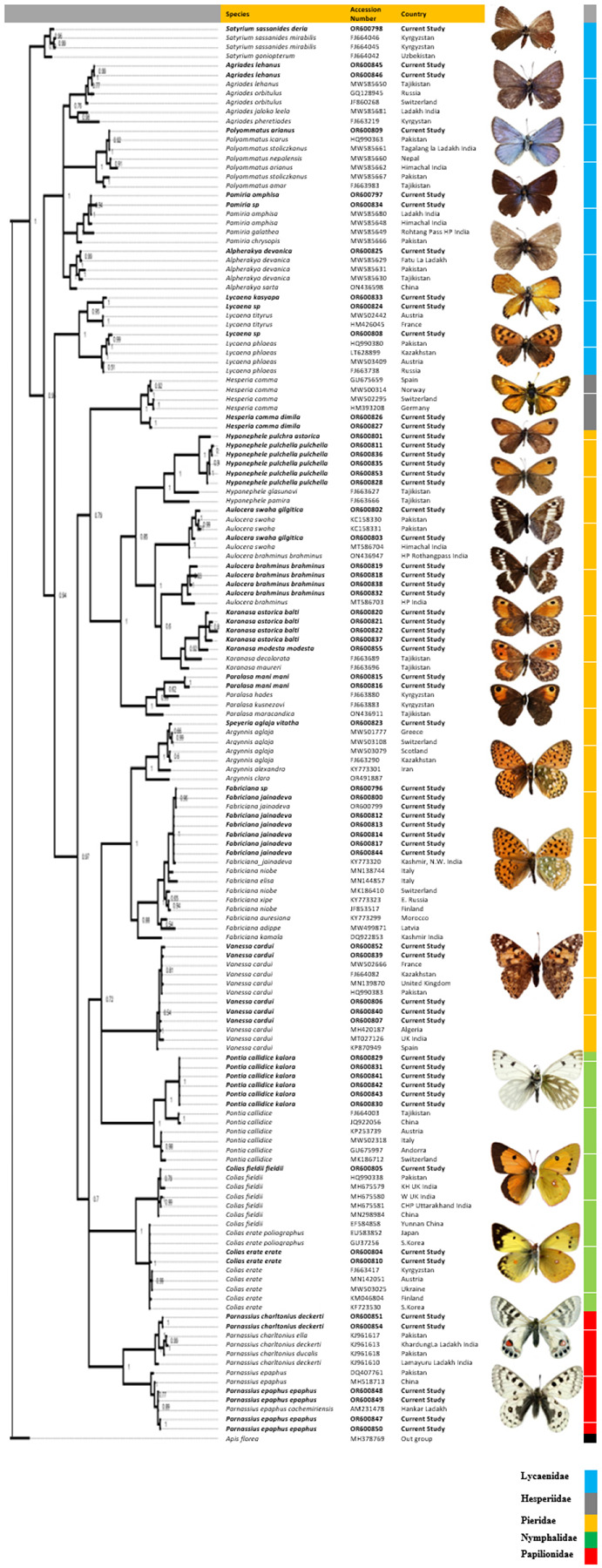
Markov Chain Monte Carlo (MCMC) Bayesian phylogenetic tree of butterflies from Ladakh Trans-Himalayas, run in MrBayes 3.2 and partitioned by two million generations. Dataset includes 60 barcodes generated in this study representing 23 species as depicted in the figure, along with 92 additional barcodes downloaded from GenBank. *Apis florea* was used as an out-group.

For the majority of the species, the distance within them was found to be less than 1.2% (Table 3), the highest observed in *Karanasa astorica* (1.21%) and *Aulocera brahminus* (1.07%). Intraspecific divergence could not be calculated for the 8 species that were represented by a single barcode. The interspecific genetic divergence among the species ranged from 4.19 – 18.54% (Table 4), except between *Hyponephele pulchra* and *H. pulchella* (2.34%), and *Karanasa astorica* and *K. modesta* (2.19%), both the cases indicating low interspecific divergence. The addition of sequences from the database had a mixed effect on the intra- and inter-species divergence values. More or less all the species showed little increase in both the divergence values, but with the exception of four species showing a considerable increase in their intraspecific divergence (marked as bold in Table 3), however their max intraspecific divergence remaining < 2.3%, thus maintaining the barcode gap. The interspecific nucleotide divergence was found to be well above the universal 3% threshold value for most of the species, even for the highly cryptic congeneric species like *Colias fieldii-C. erate* (4.2%), *Parnassius charltonius-P. epaphus* (7.3%) and *Aulocera brahminus-A. swaha* (7.4%). Thus overall, for majority of the species, a distinct barcoding gap existed within and between them, without any overlap in intra- and interspecific nucleotide divergence. On NJ cluster analysis, the generated sequences formed monophyletic clades with conspecific sequences from the database, irrespective of the barcodes being of distant geographic locations (Fig. 2). For the novel submissions, their congenerics were seen to clade closely. Bayesian analysis could also distinguish between highly cryptic species forming sister clade with each other. Among the only unresolved clades were genus *Fabriciana* and *Polyommatus*, and a mixed clade of *A. swaha* (generated in this study) and *A. brahminus* (ON436947 - downloaded from NCBI).

Of the 4 specimens that we were not able to identify, *Fabriciana sp*. (OR600796) and *Pamiria sp* (OR600834) were seen to cluster with the respective clades of *F*. *jainadeva* and *P. omphisa*. Out of the 2 *Lycaena* specimens, one (OR600824) was seen to form clade with our generated sequence for *L. kasyapa* (OR600833) with a divergence of 0.3% , whereas the other barcode (OR600824) clustered with *L. phlaeas* with a divergence value of 0.7% when compared to other L. *phlaeas* barcodes from the database. Since, *L. phlaeas* has already been recorded from Ladakh in our study [39], we identified that particular barcode as that of *L. phlaeas*.

As previous studies have indicated that nearly all phylogeny is a rather complex structure consisting of numerous nested monophyletic lineages [39], this study was also not an exception. As seen in the tree, the monophyletic cluster for *P. callidice*, with 2.3% intraspecific divergence, nests two prominent subclades, one having our generated barcodes of *P. c. kalora* forming sister clades with the barcodes from Central Asia (possibly *P. c. amaryllis* & *P. c. halasia)* having a genetic distance of 2.5%; another subclade having barcodes from Europe (possibly *P. c. callidice)* with a distance of 2.8%. Similar subcladings was also observed for the lineages of *H. comma, S. sassanides, S. aglaja, L. phlaeas* having 14, 3, 14 and 30 subspecies worldwide respectively. Our generated barcode for *S. sassanides deria* forms a subclade with those of *S. s. mirabilis from* Kyrgyzstan with a distance of 0.8% between them. For *H. comma*, generated sequences of *H. c. dimila* from Ladakh are seen to form subclades with the European barcodes (probably *H. c. comma*) having a 2.0% distance between them. However, for *Colias erate*, subclading wasn’t observed, although they have 8 subspecies. *Vanessa cardui* with no designated subspecies also lacks subcladings.

## Discussion

This study, having barcoded 22% of Ladakh’s Rhopaloceran fauna, marks the initial step towards constructing a curated DNA barcode reference library for the butterflies of Ladakh, up to subspecies level wherever possible, which is also supported by strong morphological taxonomy [40]. Barcodes of *Hyponephele pulchra astorica, H. pulchella pulchella, Lycaena kasyapa, Karanasa astorica balti, Karanasa modesta modesta and* a Schedule II species *Paralasa mani mani* were submitted to the database for the first time. BLAST and BOLD searches could identify only 50% of the generated barcode sequences correctly upto species level, the rest could be identified only up to their genus level. Few cases (*C. erate, F*. *jainadeva, P. arianus)* were observed where both BLAST and BOLD showed high similarity percentages (99–100%) but failed to identify the generated sequences up to species level, even when their sequence was already available at the database (Table S2). Taxonomic experts are usually lacking for many problematic groups and regions, and validating the taxonomy of large amounts of data is a challenge. Hence, the reliability of identity search engines alone, especially for very similar-looking organisms is not promising. Our generated barcodes mostly matched with those from distant geographical locations in the GenBank database, mostly due to the unavailability of data from the Indian subcontinent. The maximum matches were found to be with the deposited sequences from Central Asian countries (Tajikistan, Kyrgyzstan, Kazakhstan), followed by the neighboring Himalayan country (Pakistan), Indian states (Uttarakhand & Himachal Pradesh), and also with barcodes from European countries for the widely distributed Eurasian species. This highlights the awareness gap in Asian Lepidoptera research as compared with Europe. Inclusion of the geographically distant conspecific and congeneric sequences from the database resulted in an overall increase of intraspecific divergences, the results corresponding to previous studies with similar findings of increased geographical distance being often associated with an increased genetic divergence, although the increase having little to no effect on the identification of species [7,22]. However, for *Vanessa cardui*, a widely distributed Eurasian species, the divergence value (0.3%) remained almost unchanged, since long distant dispersal abilities of butterflies can result in low intraspecific divergence and shared haplotypes even from different countries [41].

On applying the 3% threshold rule for species identification [42], the species richness of Ladakh was slightly underestimated as it failed to discriminate the closely related species pairs *Hyponephele pulchra* and *H. pulchella*, along with *Karanasa astorica* and *K. modesta*. Earlier research has demonstrated that closely related congeneric Lepidoptera species typically exhibit more than 2% genetic divergence [42–43], although some sister species display lower divergence too [44–45], revealing intraspecific variations worthy of taxonomic consideration. Additionally, both of these mountainous species pairs are almost look-alike, and even their distribution is overlapping, which is restricted to the Himalayas. The reason for the low divergence value between them can be due to the interspecies hybridization among borderline species in mountainous terrains or for recently diverging lineages. *Karanasa*, a typical alpine genus having isolated populations with many of its species flying together in narrow zones of overlap, has been shown to interbreed and thus produce widely varied series of local populations even within a limited range [46]. Our sample size was low, and additionally with no representative conspecific barcodes available in the database for comparison, the actual reason for this low interspecific divergence remains debatable. Similar results have also been observed in other studies where the 3% threshold value, as well as the 10X rule, undervalued the actual species richness [22–23]. It is already established how an optimal threshold value will always be taxon-specific and a universal threshold is likely to be ineffective even within a small group [47] and only a much more fine-grained robust genetic survey for these groups can provide a better understanding of the low interspecific divergences, which can only be achieved with more barcode addition to the database.

In comparison to threshold divergences, NJ cluster analysis could accurately distinguish all the 23 morpho species, with their barcode sequences forming distinct and non-overlapping monophyletic clusters in the phylogenetic tree. Even the two most closely related species pair with the lowest intraspecies divergences, formed separate clades with strong bootstrap support in the NJ tree. Our worn-out specimens could also be identified accurately as they clustered with their conspecific barcodes, thus proving the efficacy of DNA barcoding. In most of the cases, conspecific barcodes of distant geographic areas from the database were seen to form monophyletic clades with our generated sequences, but with few exceptions. Our generated sequences for each of the two species *A. swaha* and *A. brahminus* were seen to cluster with their conspecifics from the database respectively except a single sequence of *A. brahminus* (ON436947) from the database that clustered with the *A. swaha* clade. This pair being morphologically very similar and also having overlapping distribution, lack of taxonomical expertise can easily lead to species misidentification. Also, apart from that single sequence of *A. brahminus* (ON436947), all the other conspecific sequences for the species clustered together in a separate clade than *A. swaha*. Hence we treat ON436947 as a case of misidentified submission. As discussed by others, many such misidentified entries have been reported in both GenBank and BOLD [7]. Since DNA barcoding relies heavily upon reference databases, identification becomes complicated when reference sequences for a particular species are unavailable or incorrectly identified. Thus proper precautions needs to be taken while uploading any sequence to control doubtful data and potential misidentifications.

The resulting phylogenetic tree unveiled the ancestral lineage of the subfamily Polyommatinae, encompassing *Pamiria omphisa, Polyommatus arianus, Alpherakya devanica*, and *Agriades lehanus*, constituting the most intricate cluster within the family Lycaenidae. This group diverged from the sister groups of the subfamilies Theclinae and Lycaeninae a significant amount of time ago [48]. Within Polyommatinae, the taxonomically controversial *Polyommatus* group also showed mixed clading. In the family Nymphalidae, the lineages of genus *Hyponephele, Paralasa, Aulocera* and *Karanasa* within the subfamily Satyrinae are found to diverge from species *Argynnis aglaja* and *Fabriciana jainadeva* in the subfamily Heliconiinae, as well as from *Vanessa cardui* in the subfamily Nymphalinae. The unique ecology of species might explain differences in the genetic structure and clading for the very closely related *A. aglaja* and *Fabriciana* species, as explored by Polic et al [49]. Our phylogenetic analysis of species *Pontia callidice* within the subfamily Pierinae, as well as *Colias erate* and *Colias fieldii* within the subfamily Coliadinae of the family Pieridae, revealed substantial divergence from each respective clade, similar to the findings of Wahlberg et al [50]. Notably, several prominent genera from the Trans-Himalayan region of India, such as *Polyommatus, Fabriciana, Parnassius*, and *Karanasa* still exhibit significant taxonomic gaps.

In certain species, variations were observed within the same phylogenetic clades and subclades, forming nested lineages, with significant intraspecies variation being observed among them. When genetic distances between region-specific local populations (supposing the subspecies differs with locality) for each of these individual species were calculated (Table S3 - Table S6), it was observed that the distances of Ladakh’s population differed substantially from the European-American ones than the Central Asian ones. Overall intersubspecific divergence values ranged from 0.3 – 3.8%, the highest observed between populations of *H. comma* (Table S5), followed by *P. callidice* (Table S3). These differences may be attributed to taxonomic intricacies present within the species, particularly at the subspecies level across various biogeographic ranges or other hierarchical levels of population differentiation. Most of the species that showed this trend are mostly of European origin and well resolved upto subspecies level morphologically, with number of subspecies designated for *P. callidice* being 8 that of *H*. *comma* and *S. aglaja* is 14, and *L. phlaeas* having 30 subspecies worldwide. Intersubspecific distances can range from 0.5 – 0.7%, and even as low as 0 – 0.2% for geographically closer subspecies, with well-supported clusters comprising of multiple subspecies [51]. Standard 658bp of COI has shown success in distinguishing subspecies of Malaysian butterfly [21], whereas few other butterfly studies failed to resolve the named subspecies based only on COI [51,52] and had to depend on additional genes and microsatellite markers. This difference in success rates of detecting butterfly subspecies is mainly because the intersubspecific genetic distance is likely to be small (< 2%) and even overlaps with the range of intraspecific distances at times [21], as also seen from our dataset. Historically, subspecies designation is mainly supported by the presence of consistent morphological differences between geographically isolated populations of a single species, often supplemented with their ecological data [53]. However, it has always been a subject of debate for decades [54–55], much of which is because of the inability to reach a common consensus on the minimal diagnostic standards for subspecies status. Even though in recent times, molecular data is becoming increasingly available to supplement classical morphological characters, it is alone not sufficient to diagnose a subspecies [52], mainly because majority of the barcodes deposited in GenBank and BOLD do not include subspecies names [56]. Based on locality data, it can be possible to narrow down the subspecies identity, but that information too is often missing or inaccurate for database records, thus making it more difficult to delimit subspecies. However, because taxa proposed for protection by government conservation agencies are often listed at the subspecies level [57–58], it is important to attach subspecies names to records in DNA barcode databases and define standard subspecies delimitation thresholds.

## Conclusion

The present study contributes to the ongoing global effort of building robust barcode reference libraries, enhancing the existing database for the Trans-Himalayan region of Ladakh for future studies. The usefulness of DNA barcoding as a complementary tool to traditional morphology was established, although 75% of Ladakh’s butterfly fauna awaits analysis. This library also delivers an overview of the unique genetic composition of Ladakh’s butterfly owing to potential hybridization and ongoing speciation events typical of restricted populations, also revealing cases of cryptic diversity and evolutionary significant units. To understand the evolutionary complexity of actively speciating vulnerable taxa, it is also necessary to establish threshold criteria specific to the typical mountainous species. Thus, proper expertise and precautions should be ensured for accurate species identification and verification while building the barcode libraries in order to eliminate misidentifications and confusion. Also, a common consensus should be reached for attaching subspecies name while submitting sequences to the database, so as to have a better understanding of the extent of genetic variation within populations from different geographical locations and their specific evolutionary history, especially for widely distributed species. Our study also highlights the significant gap in the butterfly molecular research in India as the genetic diversity in the global database is not that much represented as it should be, given the amount of taxonomic revisions currently undergoing. Overall, this study provides a basis for improving the understanding of the mechanisms that have shaped the genetic diversity of Ladakh’s butterfly fauna in a comprehensive manner which will ultimately ensure effective future conservation measures for these geographically restricted populations adapted to the Trans-Himalayan climate.

## Figures and Tables

**Table 1.** List of specimens analyzed in the present study. GenBank accessions, morpho-ID, collection details, and sex are provided for each of the 60 specimens from Ladakh.

GenBank Accession no	Family	Subfamily	Species	Sub Sp	Location (District, exact collection site)	Coordinates	Alt (m)	Collection Date	Sex
OR600796 ^+^	Nymphalidae	Heliconiinae	*Fabriciana jainadeva*		Kargil, Tangole	34.04866 N 75.93215 E	3795	03-Aug-19	Male
OR600797	Lycaenidae	Polyommatinae	*Pamiria omphisa*		Kargil, Tangole	34.04866 N 75.93215 E	3795	03-Aug-19	Male
OR600798	Lycaenidae	Theclinae	*Satyrium sassanides*	*deria*	Kargil, Tangole	34.04866 N 75.93215 E	3795	03-Aug-19	
OR600799	Nymphalidae	Heliconiinae	*Fabriciana jainadeva*		Kargil, Tangole	34.04866 N 75.93215 E	3795	03-Aug-19	Female
OR600800	Nymphalidae	Heliconiinae	*Fabriciana jainadeva*		Kargil, Tangole	34.04866 N 75.93215 E	3795	03-Aug-19	Female
OR600801 *	Nymphalidae	Satyrinae	*Hyponephele pulchra*	*astorica*	Kargil, Tangole	34.04866 N 75.93215 E	3795	03-Aug-19	Female
OR600802	Nymphalidae	Satyrinae	*Aulocera swaha*	*gilgitica*	Kargil, Thovina	34.37176 N 75.98422 E	3066	26-Jul-19	Male
OR600803	Nymphalidae	Satyrinae	*Aulocera swaha*	*gilgitica*	Kargil, Thovina	34.37176 N 75.98422 E	3066	26-Jul-19	Female
OR600804	Pieridae	Coliadinae	*Colias erate*	*erate*	Kargil, Thovina	34.37876 N 75.98422 E	3066	26-Jul-19	Male
OR600805	Pieridae	Coliadinae	*Colias fieldii*	*fieldii*	Kargil, Thovina	34.37176 N 75.98422 E	3066	26-Jul-19	Male
OR600806	Nymphalidae	Nymphalinae	*Vanessa cardui*		Kargil, Thovina	34.37176 N 75.98422 E	3066	26-Jul-19	Female
OR600807	Nymphalidae	Nymphalinae	*Vanessa cardui*		Kargil, Thovina	34.37176 N 75.98422 E	3066	26-Jul-19	Male
OR600808 ^+^	Lycaenidae	Lycaeninae	*Lycaena kasyapa*		Kargil, Thovina	34.37176 N 75.98422 E	3066	26-Jul-19	Male
OR600809	Lycaenidae	Polyommatinae	*Polyommatus arianus*		Kargil, Thovina	34.37176 N 75.98422 E	3066	26-Jul-19	Male
OR600810	Pieridae	Coliadinae	*Colias erate*	*erate*	Kargil, Thovina	34.37876 N 75.98422 E	3066	26-Jul-19	Male
OR600811 *	Nymphalidae	Satyrinae	*Hyponephele pulchella*	*pulchella*	Kargil, Skamboo	34.45749 N 76.24263 E	3066	29-Jul-19	Female
OR600812	Nymphalidae	Heliconiinae	*Fabriciana jainadeva*		Kargil, Skamboo	34.45749 N 76.24263 E	3066	29-Jul-19	Male
OR600813	Nymphalidae	Heliconiinae	*Fabriciana jainadeva*		Kargil, Skamboo	34.45749 N 76.24263 E	3066	29-Jul-19	Female
OR600814	Nymphalidae	Heliconiinae	*Fabriciana jainadeva*		Kargil, Skamboo	34.45749 N 76.24263 E	3066	29-Jul-19	Female
OR600815 *	Nymphalidae	Satyrinae	*Paralasa mani*	*mani*	Kargil, Skamboo	34.45749 N 76.24263 E	3066	29-Jul-19	Female
OR600816 *	Nymphalidae	Satyrinae	*Paralasa mani*	*mani*	Kargil, Skamboo	34.45749 N 76.24263 E	3066	29-Jul-19	Female
OR600817	Nymphalidae	Heliconiinae	*Fabriciana jainadeva*		Kargil, Skamboo	34.45749 N 76.24263 E	3066	29-Jul-19	Female
OR600818	Nymphalidae	Satyrinae	*Aulocera brahminus*	*brahminus*	Kargil, Sapi	34.36671 N 76.13443 E	3975	30-Jul-19	Male
OR600819	Nymphalidae	Satyrinae	*Aulocera brahminus*	*brahminus*	Kargil, Sapi	34.36671 N 76.13443 E	3975	30-Jul-19	Female
OR600820 *	Nymphalidae	Satyrinae	*Karanasa astorica*	*balti*	Kargil, Sapi	34.36671 N 76.13443 E	3975	30-Jul-19	Female
OR600821 *	Nymphalidae	Satyrinae	*Karanasa astorica*	*balti*	Kargil, Sapi	34.36671 N 76.13443 E	3975	30-Jul-19	Female
OR600822 *	Nymphalidae	Satyrinae	*Karanasa astorica*	*balti*	Kargil, Sapi	34.36671 N 76.13443 E	3975	30-Jul-19	Female
OR600823	Nymphalidae	Heliconiinae	*Speyeria aglaja*	*vitatha*	Kargil, Sapi	34.36671 N 76.13443 E	3975	30-Jul-19	Male
OR600824 ^+^	Lycaenidae	Lycaeninae	*Lycaena phlaeas*		Kargil, Sapi	34.35398 N 76.10131 E	4289	31-Jul-19	Male
OR600825	Lycaenidae	Polyommatinae	*Alpherakya devanica*		Kargil, Sapi Ree	34.35398 N 76.10131 E	4289	31-Jul-19	
OR600826	Hesperiidae	Hesperiinae	*Hesperia comma*	*dimila*	Kargil, Sapi Ree	34.35398 N 76.10131 E	4289	31-Jul-19	Male
OR600827	Hesperiidae	Hesperiinae	*Hesperia comma*	*dimila*	Kargil, Sapi Ree	34.35398 N 76.10131 E	4289	31-Jul-19	Female
OR600828 *	Nymphalidae	Satyrinae	*Hyponephele pulchella*	*pulchella*	Kargil, Sapi Ree	34.35398 N 76.10131 E	4289	31-Jul-19	Male
OR600829	Pieridae	Pierinae	*Pontia callidice*	*kalora*	Kargil, Sapi Ree	34.35398 N 76.10131 E	4289	31-Jul-19	Male
OR600830	Pieridae	Pierinae	*Pontia callidice*	*kalora*	Kargil, Sapi Ree	34.35398 N 76.10131 E	4289	31-Jul-19	Female
OR600831	Pieridae	Pierinae	*Pontia callidice*	*kalora*	Kargil, Sapi Ree	34.35398 N 76.10131 E	4289	31-Jul-19	Male
OR600832	Nymphalidae	Satyrinae	*Aulocera brahminus*	*brahminus*	Kargil, Parkachik	34.09197 N 75.96881 E	4061	02-Aug-19	Male
OR600833 *	Lycaenidae	Lycaeninae	*Lycaena kasyapa*		Kargil, Parkachik	34.09197 N 75.96881 E	4061	02-Aug-19	Male
OR600834 ^+^	Lycaenidae	Polyommatinae	*Pamiria omphisa*		Kargil, Labar	34.34182 N 75.89006 E	4308	06-Aug-19	Female
OR600835 *	Nymphalidae	Satyrinae	*Hyponephele pulchella*	*pulchella*	Kargil, Labar	34.34182 N 75.89006 E	4308	06-Aug-19	Female
OR600836 *	Nymphalidae	Satyrinae	*Hyponephele pulchella*	*pulchella*	Kargil, Labar	34.34182 N 75.89006 E	4308	06-Aug-19	Female
OR600837 *	Nymphalidae	Satyrinae	*Karanasa astorica*	*balti*	Kargil, Labar	34.34182 N 75.89006 E	4308	06-Aug-19	Male
OR600838	Nymphalidae	Satyrinae	*Aulocera brahminus*	*brahminus*	Kargil, Labar	34.34182 N 75.89006 E	4308	06-Aug-19	Male
OR600839	Nymphalidae	Nymphalinae	*Vanessa cardui*		Leh, Panamik	34.61938 N 77.52237 E	3349	11-Aug-19	Female
OR600840	Nymphalidae	Nymphalinae	*Vanessa cardui*		Leh, Panamik	34.84504 N 77.50213 E	3290	12-Aug-19	Male
OR600841	Pieridae	Pierinae	*Pontia callidice*	*kalora*	Leh, Khardung La	34.27226 N 77.61206 E	5310	13-Aug-19	Male
OR600842	Pieridae	Pierinae	*Pontia callidice*	*kalora*	Leh, Khardung La	34.27226 N 77.61206 E	5310	13-Aug-19	Male
OR600843	Pieridae	Pierinae	*Pontia callidice*	*kalora*	Leh, Khardung La	34.27226 N 77.61206 E	5310	13-Aug-19	Male
OR600844	Nymphalidae	Heliconiinae	*Fabriciana jainadeva*		Leh, Khardung La	34.27226 N 77.61206 E	5310	13-Aug-19	Male
OR600845	Lycaenidae	Polyommatinae	*Agriades lehanus*		Leh, Khardung village	34.40641 N 77.64812 E	4128	13-Aug-19	Female
OR600846	Lycaenidae	Polyommatinae	*Agriades lehanus*		Leh, Khardung village	34.40641 N 77.64812 E	4128	13-Aug-19	Male
OR600847	Papilionidae	Parnassiinae	*Parnassius epaphus*	epaphus	Leh, Khardung La	34.27226 N 77.61206 E	5310	13-Aug-19	Male
OR600848	Papilionidae	Parnassiinae	*Parnassius epaphus*	epaphus	Leh, Khardung La	34.27226 N 77.61206 E	5310	13-Aug-19	Male
OR600849	Papilionidae	Parnassiinae	*Parnassius epaphus*	epaphus	Leh, Khardung La	34.27226 N 77.61206 E	5310	13-Aug-19	Male
OR600850	Papilionidae	Parnassiinae	*Parnassius epaphus*	epaphus	Leh, Khardung La	34.27226 N 77.61206 E	5310	13-Aug-19	Male
OR600851	Papilionidae	Parnassiinae	*Parnassius charltonius*	*deckerti*	Leh, Lamayuru	34.29202 N 76.69819 E	4015	15-Aug-19	Male
OR600852	Nymphalidae	Nymphalinae	*Vanessa cardui*		Leh, Lamayuru	34.29202 N 76.69819 E	4015	15-Aug-19	Female
OR600853 *	Nymphalidae	Satyrinae	*Hyponephele pulchella*	*pulchella*	Leh, Lamayuru	34.29202 N 76.69819 E	4015	15-Aug-19	Female
OR600854	Papilionidae	Parnassiinae	*Parnassius charltonius*	*deckerti*	Leh, Lamayuru	34.29202 N 76.69819 E	4015	15-Aug-19	Male
OR600855 *	Nymphalidae	Satyrinae	*Karanasa modesta*	*modesta*	Kargil, Tangole	34.04866 N 75.93215 E	3795	03-Aug-19	Female

+ Specimens that could be assigned only up to genus level through morpho-taxonomy, later assigned to respective species using molecular taxonomy

* Novel submissions to the database

**Table 2.** Average Nucleotide composition of the generated COI sequences, the base composition exhibiting a bias towards Adenine and Thymine with a combined total of 70.2%, typical of invertebrate genes

Codon Position	Emperical Base frequencies (%)			
	T	C	A	G
All	39.9	15.4	30.3	14.4
First	28	14.7	31.5	25.4
Second	43	24.5	15.4	16.7
Third	48	7.2	44.1	1.0

**Table 3.** Intra-Specific Mean Genetic Divergence of the generated sequences. On addition of conspecific sequences from the database little increase in divergence values was noted, except for those marked in bold showing a considerable increase in their divergence

Species	Generated barcodes	Global barcodes
*Parnassius epaphus*	0.001068377	0.003677626
*Parnassius charltonius*	0	0.007742029
***Pontia callidice***	0	**0.023247472**
*Colias fieldii*	n/c	0.003006197
*Colias erate*	0.001602565	0.001717587
*Vanessa cardui*	0.003370931	0.003275172
*Hyponephele pulchra*	n/c	n/c
*Hyponephele pulchella*	0.002245396	n/c
*Paralasa mani*	0.004815426	n/c
*Aulocera swaha*	0.008053626	0.007091219
***Aulocera brahminus***	0.010750928	**0.01689717**
*Fabriciana jainadeva*	0.000858517	0.000763126
*Speyeria aglaja*	n/c	0.003573076
*Karanasa astorica*	0.012133313	n/c
*Agriades lehanus*	0	0.001068377
***Polyommatus arianus***	n/c	**0.012966691**
*Pamiria omphisa*	0.00642768	0.003215459
*Alpherakya devanica*	n/c	0.008873866
*Lycaena kasyapa*	n/c	n/c
*Satyrium sassanides*	n/c	0.005906747
***Hesperia comma***	0.001602565	**0.012783793**
*Karanasa modesta*	n/c	n/c

**Table 4.** Inter-specific mean genetic distance based on COI gene for the sequences generated in this study along with additional sequences used from the database; The lowest and highest genetic divergences are marked in bold, observed between *Colias erate - C. fieldii* (4.19%) and *Polyommatus arianus -Hyponephele pulchra* (18.55%), respectively

Species	Parnassius epaphus	Parnassius charltonius	Pontia callidice	Colias fieldii	Colias erate	Vanessa cardui	Hyponephele pulchra	Hyponephele pulchella	Paralasa mani	Aulocera swaha	Aulocera brahminus	Fabriciana jainadeva	Speyeria aglaja	Karanasa astorica	Karanasa modesta	Agriades lehanus	Polyommatus arianus	Pamiria omphisa	Alpherakya devanica	Lycaena kasyapa	Lycaena phlaes	Satvrium sassanides
*Parnassius epaphus*																						
*Parnassius charltonius*	0.0734																					
*Pontia callidice*	0.1479	0.1647																				
*Colias fieldii*	0.1438	0.1523	0.1667																			
*Colias erate*	0.1368	0.1336	0.1639	**0.0419**																		
*Vanessa cardui*	0.1480	0.1549	0.1509	0.1430	0.1469																	
*Hyponephele pulchra*	0.1451	0.1516	0.1725	0.1673	0.1581	0.1838																
*Hyponephele pulchella*	0.1544	0.1511	0.1715	0.1604	0.1587	0.1778	0.0234															
*Paralasa mani*	0.1700	0.1776	0.1760	0.1798	G.1619	0.1671	0.1259	0.1290														
*Aulocera swaha*	0.1739	0.1832	0.1640	0.1598	0.1625	0.1591	0.1267	0.1223	0.1249													
*Aulocera brahminus*	0.1546	0.1608	0.1537	0.1671	0.1590	0.1313	0.1218	0.1223	0.1224	0.0750												
*Fabriciana jainadeva*	0.1336	0.1388	0.1342	0.1457	0.1424	0.1373	0.1324	0.1315	0.1717	0.1523	0.1304											
*Speyeria aglaja*	0.1227	0.1454	0.1457	0.1443	0.1440	0.1321	0.1461	0.1451	0.1754	0.1597	0.1391	0.01576										
*Karanasa astorica*	0.1715	0.1813	0.1664	0.1718	0.1822	0.1610	0.1362	0.1239	0.1235	0.0931	0.0820	0.1544	0.1651									
*Karanasa modesta*	0.1696	0.1763	0.1597	0.1730	0.1758	0.1601	0.1362	0.1280	0.1202	0.0913	0.0812	0.1515	0.1626	0.0219								
*Agriades lehanus*	0.1534	0.1497	0.1710	0.1590	0.1544	0.1319	0.1811	0.1802	0.1930	0.1664	0.1548	0.1621	0.1703	0.1682	0.1743							
*Polyommatus arianus*	0.1697	0.1769	0.1794	0.1612	0.1583	0.1390	**0.1855**	0.1841	0.1933	0.1798	0.1708	0.1567	0.1611	0.1714	0.1807	0.0693						
*Pamiria omphisa*	0.1633	0.1473	0.1597	0.1643	0.1559	0.1370	0.1823	0.1727	0.1805	0.1626	0.1480	0.1506	0.1664	0.1664	0.1750	0.0585	0.0802					
*Alpherakya devanica*	0.1512	0.1417	0.1626	0.1273	0.1226	0.1251	0.155S	0.1548	0.1616	0.1315	0.1354	0.1452	0.1475	0.1526	0.1576	0.0481	0.0678	0.0534				
*Lycaena kasyapa*	0.1429	0.1392	0.1431	0.1433	0.1377	0.1502	0.1502	0.1449	0.1696	0.1549	0.1322	0.1412	0.1446	0.1567	0.1659	0.1097	0.1176	0.1011	0.0890			
*Lycaena phlaes*	0.1524	0.1630	0.1526	0.1529	0.1505	0.1543	0.1703	0.1637	0.1584	0.1622	0.1501	0.1523	0.1424	0.1695	0.1782	0.1170	0.1206	0.1121	0.0870	0.0543		
*Satvrium sassanides*	0.1223	0.1487	0.1373	0.1548	0.1520	0.1495	0.1602	0.1523	0.1706	0.1574	0.1560	0.1409	0.1267	0.1647	0.1714	0.0864	0.0959	0.0863	0.0739	0.0926	0.0963	
*Hesperia comma*	0.1463	0.1637	0.1788	0.1562	0.1515	0.1545	0.1677	0.1732	0.1491	0.1508	0.1577	0.1549	0.1521	0.1700	0.1645	0.1384	0.1547	0.1506	0.1327	0.1494	0.1575	0.1144

## Data Availability

All the COI sequences generated in this study are deposited in the GenBank database of NCBI under accession numbers OR600796 - OR600855, in private mode. However, on publication, these will be released to public. The specimens are deposited in the headquarters of Zoological Survey of India, Prani Vigyan Bhawan, Kolkata-700053, and West Bengal, India.
